# Extraction, purification and antioxidant activity of flavonoids from *Potentilla anserina L*

**DOI:** 10.3389/fbioe.2025.1691606

**Published:** 2025-11-19

**Authors:** Dandan Gao, Xuankang Yang, Danqi Lu, Chenchen Wang, Jiwen Wang, Huaxin Li, Xingchen Guo

**Affiliations:** 1 Key Laboratory of Biotechnology and Bioengineering of State Ethnic Affairs Commission, Biomedical Research Center, Northwest Minzu University, Lanzhou, China; 2 College of Life Sciences and Engineering, Northwest Minzu University, Lanzhou, China

**Keywords:** *Potentilla anserina L*., ultrasound-assisted extraction, flavonoids, purification, antioxidant activity

## Abstract

**Introduction:**

*Potentilla anserina L.*, a traditional Chinese medicinal herb, is valued for its edible, medicinal, and ornamental properties.

**Methods:**

In this study, flavonoids were extracted from *Potentilla anserina L.* using ultrasonic-assisted extraction. The extraction process was optimized through response surface methodology, followed by separation and purification using Sephadex G-100 gel chromatography. Finally, the antioxidant activity of the extracted flavonoids was evaluated.

**Results:**

The results showed that the optimal extraction conditions were 60% ethanol concentration, an ultrasonic temperature of 50°C, ultrasonic power of 400 W, and ultrasonic time of 180 min. Under these conditions, the average extraction yield of flavonoids from *Potentilla anserina L.* was 3.74 ± 0.06 mg/g. The crude flavonoid extract was purified by Sephadex G-100 gel chromatography, yielding two fractions, LF-1 and LF-2, which accounted for 63.34% and 25.79% of the crude extract, respectively.

**Discussion:**

The results of in vitro antioxidant activity experiments demonstrated that both fractions (LF-1 and LF-2) exhibited significant antioxidant activity, showing a dose-dependent capacity. These findings provide a theoretical basis for the further development and utilization of flavonoids from *Potentilla anserina L..*

## Introduction

*Potentilla anserina L.,* a perennial herbaceous plant in the genus “Potentilla” of the family Rosaceae, is also known as “poke ma” in Tibetan (Guo et al., 2023). This species is not only found in alpine meadows, but also inhabits riverbanks, roadsides, and hillside grasslands, occurring at altitudes ranging from 500 to 4,100 m (Luan et al., 2022). Widely distributed across North America, Asia, and Europe. *Potentilla anserina L.* is primarily found within China’s territorial boundaries in Qinghai Province, the Tibet Autonomous Region, Gansu Province, and Sichuan Province (Yang et al., 2021). Studies have shown that this plant possesses considerable value in terms of edibility, medicinal properties, and ornamental use, indicating significant potential for further development and application (Tang et al., 2022).

*Potentilla anserina L.* is rich in nutritional and bioactive components, including polysaccharides, triterpenoids, and flavonoids. These compounds not only provided with abundant nutritional value but also exhibit a range of biological activities, such as antioxidant, anti-myocardial ischemia, antiviral, and immunomodulatory effects, thereby offering protective benefits to human organs (Čižmárová et al., 2023; Kim et al., 2020). To date, extensive studies have reported on the extraction of, polysaccharides, polyphenols (Huang et al., 2020), and other bioactive substances from *Potentilla anserina L*. Flavonoids, in particular, are widely recognized for their diverse functionalities, including antihypertensive, antibacterial, anti-inflammatory, and antioxidant properties (Binkowska, 2020). Therefore, this study is dedicated to optimizing the ultrasound-assisted extraction process of flavonoids from *Potentilla anserina L.* and characterizing the *in vitro* antioxidant activity of the resulting flavonoid extracts.

Flavonoids are traditionally extracted from natural plants using solvent extraction, a technique that exploits differences in the solubility of target compounds across various solvents to achieve separation (Tang et al., 2023). This method is known for its simple equipment requirements, ease of operation, and relatively low cost. However, it also has drawbacks, such as potential environmental pollution caused by solvent usage and the need for multiple extraction cycles. To improve the efficiency of natural product extraction, several emerging techniques have been developed and applied, including silica gel column chromatography, methanol Soxhlet extraction, and ultrasonic-assisted extraction and supercritical carbon dioxide extraction. Despite being recognized for its green and safe profile along with high extraction efficiency, supercritical CO_2_ extraction is characterized by high equipment costs, elevated energy consumption, and a requirement for stringent raw material pretreatment. Among these, ultrasonic-assisted extraction stands out due to its short extraction time, cost-effectiveness, and environmental friendliness, which have contributed to its widespread use in the extraction of bioactive compounds from plant materials. The mechanism of ultrasonic-assisted extraction relies on the mechanical effects, cavitation effects, thermal effects, and secondary effects induced by ultrasonic waves. These effects enhance the penetration ability of the solvent and accelerate the release and diffusion of target molecules into the solvent, thereby improving overall extraction efficiency (Zhang et al., 2018).

In this study, the ultrasonic-assisted extraction method was employed to extract flavonoids from *Potentilla anserina L.* single-factor experiments and response surface methodology were utilized to optimize the extraction conditions. Subsequently, Sephadex gel chromatography was used to separate and purify the flavonoids from the extracted solution. Finally, the *in vitro* antioxidative activity of the purified flavonoids was evaluated by assessing their DPPH radical scavenging capacity, hydroxyl radical (·OH) scavenging activity, superoxide anion (·O_2_^−^) scavenging ability, and total reducing power. These assays provide a comprehensive understanding of the potential antioxidant properties of *Potentilla anserina L.* flavonoids, which may contribute to enhancing the utilization value of this plant in pharmaceutical, nutraceutical, and functional food applications.

## Materials and methods

### Materials and reagents

Analytical grade ethanol, vitamin C (V_C_), and sodium nitrite (NaNO_2_) were purchased from Chengdu Cologne Chemical Co., Ltd. (Chengdu, China). Rutin (>98%) and 1,1-diphenyl-2-picrylhydrazyl (DPPH) were obtained from Aladdin Reagent Co., Ltd. (Shanghai, China). Aluminum nitrate (Al(NO_3_)_3_) and sodium hydroxide (NaOH) were analytical grade and provided by the Beijing Soleibao Technology Co., Ltd. (Beijing, China).

*Potentilla anserina L.* was purchased from a local market in Lanzhou, Gansu Province, China. The original collection site was Hezuo City, Gannan Tibetan Autonomous Prefecture (102°53′E, 34°57′N), with samples collected in 2025. It was cleaned and then freeze-dried using a vacuum freeze-dryer (LGJ-100F, Thermo, United States). The dried *Potentilla anserine L.* was ground into powder, which was collected through an 80-mesh sieve. The powder was subsequently degreased twice with n-hexane (M: V = 1:3 g/mL) for 6 h each time was conducted under room temperature conditions, dried, and stored for later use.

### Ultrasound-assisted extraction of flavonoids

A total of 3.00 g of defatted *Potentilla anserina L* powder was accurately weighed, and 60 mL of ethanol was added according to a solid–liquid ratio of 1: 20 (g/mL). The extraction was performed using an ultrasonic cleaner (SB-500DTY, Ningbo Xinzhi Biotechnology Co., China), and the procedure was repeated three times. The combined extracts were centrifuged at 5,000 rpm for 10 min (Heraeus Multifuge X1R, Thermo, United States). Following concentration under vacuum rotary evaporation, the crude flavonoids obtained must be stored at 4 °C in complete darkness within amber glass containers to prevent ultraviolet light from causing oxidation and degradation of the flavonoid structure (Yu et al., 2018).

### Determination of flavonoid extraction rate

The yield of flavonoids was determined using the colorimetric method with aluminium nitrate (Liao et al., 2021). A 0.5 mL aliquot of the sample was diluted with 70% ethanol to a final volume of 5 mL. Subsequently, 0.3 mL of 5% NaNO_2_ solution was added, and the mixture was allowed to react for 6 min. Then, 1 mL of 10% Al(NO_3_)_3_ solution was introduced, followed by a further 6 min reaction period. Finally, 10 mL of 4% NaOH solution was added, and the mixture was thoroughly mixed and left to react completely. The solution was then left at room temperature for 15 min, after which the absorbance was measured at 510 nm using a UV spectrophotometer (Nie et al., 2019). The flavonoid yield was expressed as rutin equivalent (RE) per gram of *Potentilla anserina L.* extract and calculated using the following Equation 1:

$$\text{Extraction rate}\left(\text{mg}/g\right)=\frac{\text{mass}\;\text{of}\;\text{the}\;\text{crude}\;\text{flavonoids}}{\text{mass}\;\text{of}\;\text{the}\;Potentilla\;anserina\;L.\;\text{powder}}\times 100$$ (1)

### The effect of ethanol concentration, ultrasonic temperature, ultrasonic power, and extraction time on flavonoid extraction rate

The effects of four factors on the efficiency of ultrasonic-assisted extraction were investigated to optimize the extraction yield of *Potentilla anserina L.* flavonoids. The four independent variables included ethanol concentration (50%, 60%, 70%, 80%, and 90%), ultrasonic temperature (40, 50, 60, 70, and 80 °C), ultrasonic power (250, 300, 350, 400, and 450 W), and ultrasonic time (1, 2, 3, 4, and 5 h).

### Optimization of the extraction conditions

Based on the results of single -factor experiments, the response surface methodology was employed to optimize the ultrasound-assisted extraction conditions of *Potentilla anserina L.* flavonoids. The extraction yield (Y) was selected as the response variable, while ethanol concentration (X_1_), ultrasonic power (X_2_), and ultrasonic temperature (X_3_) were chosen as the three independent variables. A total of 17 experimental runs were performed using a three-factor, three-level Box-Behnken Design (BBD), including 5 center points and 12 factorial points (−1, 0, and 1). The test factors and their levels are presented in Table 1. The experimental data were fitted to a second-order polynomial regression model described by the following Equation 2:

$$Y=Z_{0}+\sum_{i=1}^{k=3}Z_{i}X_{i}+\sum_{i=1}^{k=3}Z_{\text{ii}}X_{i}^{2}+\sum_{i=1}^{k=3}Z_{\text{ij}}X_{i}X_{j}$$ (2)

**TABLE 1:** Response surface test factors and levels.

Level	Factor		
	A ethanol concentration/%	B ultrasonic temperature/°C	C ultrasonic power/W
−1	50	40	350
0	60	50	400
1	70	60	450

Where Y is the response variable representing the extraction yield of *Potentilla anserina L.* flavonoids (%); Z_0_, Z_i_, Z_ii_, and Z_ij_ are the regression coefficients for the intercept, linear terms, quadratic terms, and interaction terms, respectively; and X_i_ and X_j_ are the coded independent variables (i ≠ j).

### Sephadex G-100 gel chromatography

The Sephadex G-100 gel chromatography system used for the separation and purification of *Potentilla anserina L.* flavonoids consists primarily of a chromatographic column unit and a detection unit. The separation unit includes a 1.0 cm × 100 cm chromatographic column (Shanghai Huxi Analytical Instrument Factory Co., Ltd.), a CXG-1 Computer-Controlled Thermostatic Chromatography Cabinet (Shanghai Jiapeng Technology Co., Ltd.), and an HL-2S Constant Flow Pump (Shanghai Huxi Analytical Instrument Factory Co., Ltd.). The detection unit comprises an HD-3 UV Detector, an HD-A Computer-Controlled Chromatography Data Acquisition System, and an SBS-100 Automatic Fraction Collector, all supplied by Shanghai Jiapeng Technology Co., Ltd.

A 10 mg/mL solution of *Potentilla anserina L.* flavonoids was filtered through a 0.45 μm microporous membrane to prevent potential clogging of the gel matrix and then loaded onto the column. Equilibration was performed using a column (1.6 cm inner diameter × 50 cm length, i. d. × L) and 8 column volumes of buffer. A loading volume of 3 mL was used, followed by isocratic elution with 0.02 mol/L phosphate buffer (pH 6.8) at a flow rate of 0.4 mL/min. The eluate was monitored at 510 nm using a UV detector, and fractions were automatically collected at intervals of 7.5 min per tube. Following multiple cycles, distinct fractions were aliquoted, lyophilized, and stored as dry powders for further analysis.

### *In vitro* antioxidant activity assay

#### Determination of DPPH radical scavenging activity

The DPPH radical scavenging capacity was evaluated using the method described by Franco et al. (2019). Various concentrations (0.1, 0.2, 0.3, 0.4, and 0.5 mg/mL) of *Potentilla anserina L.* flavonoids (2 mL each) were mixed with 2 mL of DPPH solution (0.2 mM), and the mixture was allowed to react for 30 min at room temperature in the dark. The absorbance of the resulting solution was measured at 517 nm. Control 1 consisted of 2.0 mL DPPH solution and 2.0 mL distilled water, while Control 2 consisted 2.0 mL sample solution and 2.0 mL distilled water, The Vitamin C (V_C_) group was used as the positive control, and the procedure was repeated three times. The DPPH radical scavenging activity was calculated using the following Equation 3:

$$E=\frac{A_{1}‐\left(A_{x}‐A_{2}\right)}{A_{1}}\times 100$$ (3)

Where E represents the DPPH radical scavenging activity (%); A_x_ is the absorbance of the sample group; A1 denotes the absorbance of the control 1, A_2_ refers to the absorbance of control 2.

### Determination of hydroxyl (·OH) radical scavenging activity

The hydroxyl radical (·OH) scavenging activity of flavonoids from *Potentilla anserina L.* was evaluated using the methods described by Zhou et al. (Zhou et al., 2021) and Liang et al. (Liang et al., 2018). Solutions of *Potentilla anserina L.* flavonoids were prepared at concentrations of 0.1, 0.2, 0.3, 0.4, and 0.5 mg/mL. Each sample solution (1.0 mL) was mixed with o-phenanthroline ethanol solution (0.75 mM) and FeSO_4_ solution (0.75 mM), and the mixture was incubated in a water bath at 37 °C for 30 min. Subsequently, 1.0 mL of H_2_O_2_ (0.01%) and 2.0 mL of PBS (pH 7.4) were added, followed by thorough mixing and further incubation under the same conditions for 15 min. After cooling to room temperature, the absorbance (A_x_) of each sample solution was measured at 510 nm against a blank control consisting of distilled water. Vitamin C (V_C_) solutions at the same concentrations served as a positive control, and the procedure was repeated three times. The hydroxyl radical scavenging activity was calculated using the following Equation 4:

$$E=\frac{A_{0}‐\left(A_{x}‐A_{j}\right)}{A_{0}}\times 100$$ (4)

Where A_0_ is the absorbance of the reaction system without the hydroxyl radical (OH), Ax represents the absorbance of the sample group, and Aj denotes the absorbance of the blank control.

### Determination of hydroxyl (O_2_^−^) radical scavenging activity

The superoxide anion (O_2_^−^) radical scavenging activity of *Potentilla anserina L.* flavonoids was evaluated using a previously described method. Solutions of *Potentilla anserina L.* flavonoids and vitamin C (V_C_) were prepared at concentrations of 0.1, 0.2, 0.3, 0.4 and 0.5 mg/mL, respectively. A 1.0 mL *aliquot of each flavonoid solution was mixed with* 4.5 mL of Tris-HCl buffer (pH 8.2), and the mixture was incubated in a water bath at 25 °C for 10 min. Subsequently, 0.4 mL of 25 mM catechol solution was added, the mixture was thoroughly mixed and incubated again at 25 °C for 5 min. Finally, 1 mL of 8 M hydrochloric acid was introduced to terminate the reaction, and the absorbance was measured at 320 nm. Distilled water substituted for the flavonoid solution served as the blank control, while the V_C_ group was used as the positive control, and the procedure was repeated three times. The scavenging activity was calculated using the following Equation 5:

$$E=\frac{A_{C}‐\left(A_{S}‐A_{0}\right)}{A_{C}}\times 100$$ (5)

Where A_c_ represents the absorbance of the blank control; A_s_ is the absorbance of the sample, and A_0_ denotes the absorbance of the sample solution mixed with Tris-HCl buffer after incubation.

### Assay of total reducing ability

The total reducing capacity of *Potentilla anserina L.* flavonoids was assessed using the method described in a previous study (Goswami et al., 2020). Solutions of *Potentilla anserina L.* flavonoids and vitamin C (V_C_) were prepared at concentration gradients of 2, 4, 6, 8, and 10 mg/mL, respectively. A 1 mL aliquot of each sample solution was mixed with 2.5 mL of phosphate buffer (pH 6.6) and 2.5 mL of 1% potassium ferricyanide solution, followed by incubation in a water bath at 50 °C for 20 min. Afterward, 2.5 mL of 10% trichloroacetic acid solution was added, and the mixture was centrifuged at 5,000 rpm for 10 min. Then, 5 mL of the resulting supernatant was combined with 5 mL of deionized water and 1 mL of 0.1% ferric chloride solution, and the reaction was allowed to proceed for 10 min. Absorbance was measured at 700 nm using deionized water as a blank reference, with V_C_ used as the positive control.

### Statistical analysis

SPSS 25.0 software was employed for data analysis, with results presented as mean ± standard deviation. A one-way analysis of variance (ANOVA) was conducted to assess statistical significance. Design-Expert software (Version 8.0.6, Stat-Ease, Inc., Minneapolis, MN, United States) was utilized for experimental design. All experiments and analyses were performed in triplicate to ensure reproducibility. The 50% inhibitory concentrations (IC_50_ values) were calculated using the probit analysis method with SPSS software.

## Results

### The effect of ethanol concentration, ultrasonic temperature, ultrasonic power, and extraction time on flavonoid extraction rate

**FIGURE 1:**
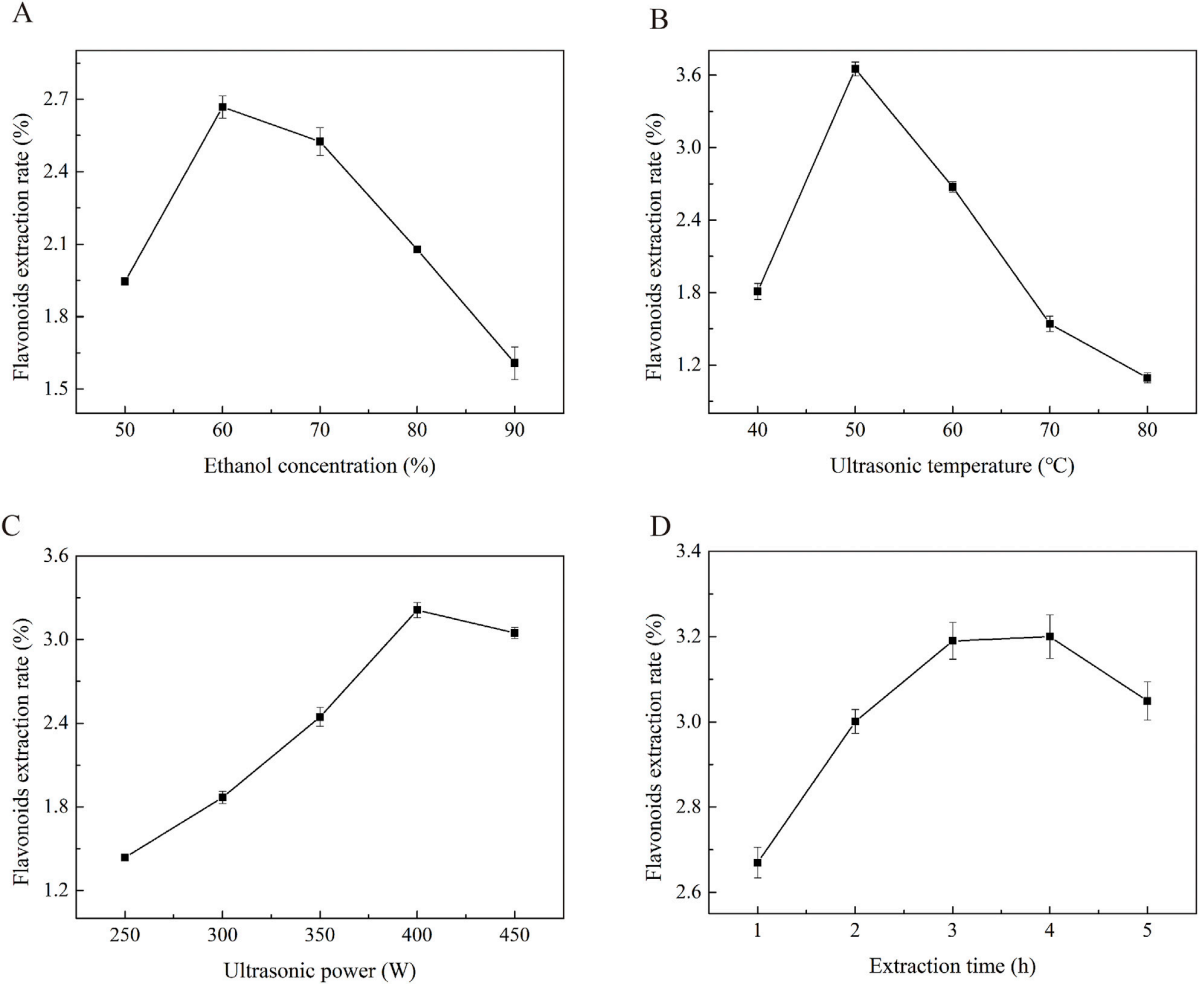
The effects of ethanol concentration **(A)**, Ultrasound temperature **(B)**, and Ultrasonic power **(C)**, Ultrasonic time **(D)** on the yield of *Potentilla anserine L*. flavonoids.

Ethanol was selected as the extraction solvent due to its high safety profile and environmental compatibility. The concentration of ethanol plays a crucial role in the extraction efficiency plant flavonoids. Flavonoids with higher polarity are more effectively extracted using low-concentration ethanol, whereas nonpolar free flavonoids are better dissolved and extracted by high-concentration ethanol. As illustrated in Figure 1A, in the single-factor experiments, all other conditions were fixed as follows: ultrasonic power of 350 W, ultrasonic temperature of 50 °C, and ultrasonic time of 3 h. The maximum flavonoid yield of 2.67 ± 0.06 mg/g was achieved when the ethanol concentration was 60%. According to the principle of like dissolves like, the solubility of flavonoid compounds in ethanol is closely related to their respective polarities. The polarity of *Potentilla anserina L.* flavonoids is comparable to that of a 60% ethanol solution, which explains the highest yield observed at this concentration. When the ethanol concentration exceeds 60%, the extraction yield decreases. A possible explanation for this decline is that excessively high ethanol concentrations may dissolve other alcohol-soluble impurities, thereby interfering with the accurate determination of flavonoid content (Michalaki et al., 2023).

The diffusion coefficient and solubility of flavonoids in the extraction solvent are improved at higher temperatures, which may contribute to an increased extraction rate (Cui et al., 2022). To evaluate the effect of ultrasonic temperature (40, 50, 60, 70, and 80 °C) on the extraction yield, experiments were conducted under fixed conditions: ultrasonic power of 350 W, ethanol concentration of 60%, and ultrasonic time of 3 h. As shown in Figure 1B, the extraction yield of *Potentilla anserina L.* flavonoids increased from 1.81% to 3.65% as the ultrasonic temperature rose from 40 °C to 50 °C, followed by a gradual decrease when the temperature was further increased to 80 °C. This trend can be explained by the fact that increasing temperature enhances the dissolution rate of flavonoids, thereby improving the extraction yield (Wu et al., 2021). However, beyond a certain threshold, excessive heat may compromise the molecular structure of flavonoids, reduce their thermal stability, and lead to degradation, ultimately resulting in a decline in yield.

The extraction of flavonoids is strongly influenced by the ultrasonic power, a critical parameter. As ultrasonic power increase, flavonoids are released and extracted through the combined effects of the mechanical forces and cavitation generated by ultrasound, which facilitates the extraction process (Chen et al., 2022). To investigate the effect of ultrasonic power (250, 300, 350, 400, and 450 W) on extraction yield, experiments were conducted under fixed conditions: an ultrasonic temperature of 50 °C, extraction time of 3, hours and ethanol concentration of 60%. As shown in Figure 1C, the flavonoid yield increased with rising power within the range of 250–400 W, reaching a maximum of 3.21 ± 0.08 mg/g at 400 W. Beyond this level, the yield decreased. This decline can be attributed to the fact that excessive ultrasonic power may induce intense cavitation activity, leading to structural degradation of flavonoids and consequently reducing the extraction yield (Xue Y. et al., 2022).

Ultrasonic treatment enhances the extraction efficiency of flavonoids by disrupting the chemical bonds of polysaccharides in cell walls through high pressure, elevated temperature, and shear forces generated during sonication (Chen et al., 2017). To evaluate the effect of ultrasonic time (1, 2, 3, 4, and 5 h) on extraction yield, experiments were conducted under fixed conditions: an ultrasonic temperature of 50 °C, ultrasonic power of 350 W, and ethanol concentration of 60%. As shown in Figure 1D, the maximum flavonoid yield of 3.20 ± 0.02 mg/g was achieved when the ultrasonic time was set to 4 h. Beyond this point, the extraction yield decreased significantly. This decline can be attributed to the fact that although the amount of dissolved flavonoids increases gradually with extraction time (Xue H. et al., 2022), the rate of increase becomes negligible after 3 h, as most flavonoids have already been extracted. Prolonged exposure beyond 4 h may lead to structure degradation of the flavonoids, resulting in a reduced yield.

### Optimization of flavonoid extraction conditions by response surface methodology

#### Statistical analysis and the model fitting

According to the principles of the Box-Behnken Design (BBD) in central composite experimentation, a total of 17 experimental groups were designed using Design-expert 8.0.6 software. The levels and corresponding results of the independent variables are summarized in Table 2. The extraction yield ranged from 2.58% to 3.81%, with the maximum yield achieved under the conditions of 60% ethanol concentration, 50 °C ultrasonic temperature, and 400 W ultrasonic power. A second-order polynomial regression equation was established to describe the relationship between the three independent variables and the response variable as follows:

$$Y=3.74+0.034X_{1}+0.13X_{2}+0.054X_{3}-0.02X_{1}X_{2}-0.0025X_{1}X_{3}-0.06X_{2}X_{3}-0.52X_{1}^{2}-0.46X_{2}^{2}-0.38X_{3}^{2}$$

**TABLE 2:** Box-Behnken experimental design and results of flavonoids extracted from *Potentilla anserine L.*

Test no.	Factor			Flavonoid yield/%
	A ethanol concentration/%	B ultrasonic temperature/°C	C ultrasonic power/W	
1	50	40	400	2.58
2	70	40	400	2.69
3	50	60	400	2.89
4	70	60	400	2.92
5	50	50	350	2.77
6	70	50	350	2.84
7	50	50	450	2.87
8	70	50	450	2.93
9	60	40	350	2.66
10	60	60	350	3.04
11	60	40	450	2.90
12	60	60	450	3.04
13	60	50	400	3.73
14	60	50	400	3.73
15	60	50	400	3.79
16	60	50	400	3.66
17	60	50	400	3.81

Where Y represents the flavonoid yield (%) from *Potentilla anserine L.*; X_1_, X_2_ and X_3_ denote the coded values of ethanol concentration (%), ultrasonic temperature (°C), and ultrasonic power (W), respectively.

The results of the analysis of variance (ANONA) are presented in Table 3. The model exhibited a high F-value (167.91) and a low P-value (<0.0001), indicating that it is statistically significant and reliable. The coefficient of determination (R^2^) was 0.9954, meaning that 99.54% of the variation in the response can be explained by the model, while only 0.46% remains unexplained. The adjusted R^2^ value (R^2^Adj = 0.9895), which is close to R^2^, further confirms that the model fits the experimental data well and demonstrates a strong correlation between the observed and predicted values (Li et al., 2020). Additionally, the low coefficient of variation (C.V. = 1.45%) indicates high precision and reliability of the model. As shown in Table 3, among the factors included in the regression equation, the linear term X_2_ showed an extremely significant effect, X_3_ was significant, followed by the interaction term X_2_X_3_, which was also significant. The quadratic terms X_1_^2^, X_2_^2^, and X_3_^2^ were all extremely significant. The order of influence on the flavonoid extraction yield from *Potentilla anserina L.* was determined as X_2_ > X_3_ > X_1_, indicating that ultrasonic temperature had the greatest impact on the extraction rate., followed by ultrasonic power, and finally ethanol concentration (Liu et al., 2019).

**TABLE 3:** Indigenous analysis of regression equation coefficient.

Sources of variance	Degrees of freedom	Sum of squares	Mean square	*F value*	P value
A	1	0.009113	0.009113	4.46	0.0725
B	1	0.14	0.14	68.78	<0.0001**
C	1	0.023	0.023	11.32	0.0127*
AB	1	0.0016	0.0016	0.78	0.4902
AC	1	0.000025	0.000025	0.012	0.9328
BC	1	0.014	0.014	7.05	0.0348*
A^2^	1	1.12	1.12	548.44	<0.0001**
B^2^	1	0.88	0.88	432.97	<0.0001**
C^2^	1	0.59	0.59	291.10	<0.0001**
Model	9	3.09	0.34	167.91	<0.0001**
Residual error	7	0.014	0.00204		
Lack of fit	3	0.000375	0.000125	0.036	0.9895
Pure error	4	0.014	0.00348		
Cor total	16	3.10			
	R_2_ = 0.9954	R^2^_Adj_ = 0.9895		C.V.% = 1.45	

### Response surface and contour plot analyses of the extracted *Potentilla anserina L.* flavonoids

**FIGURE 2:**
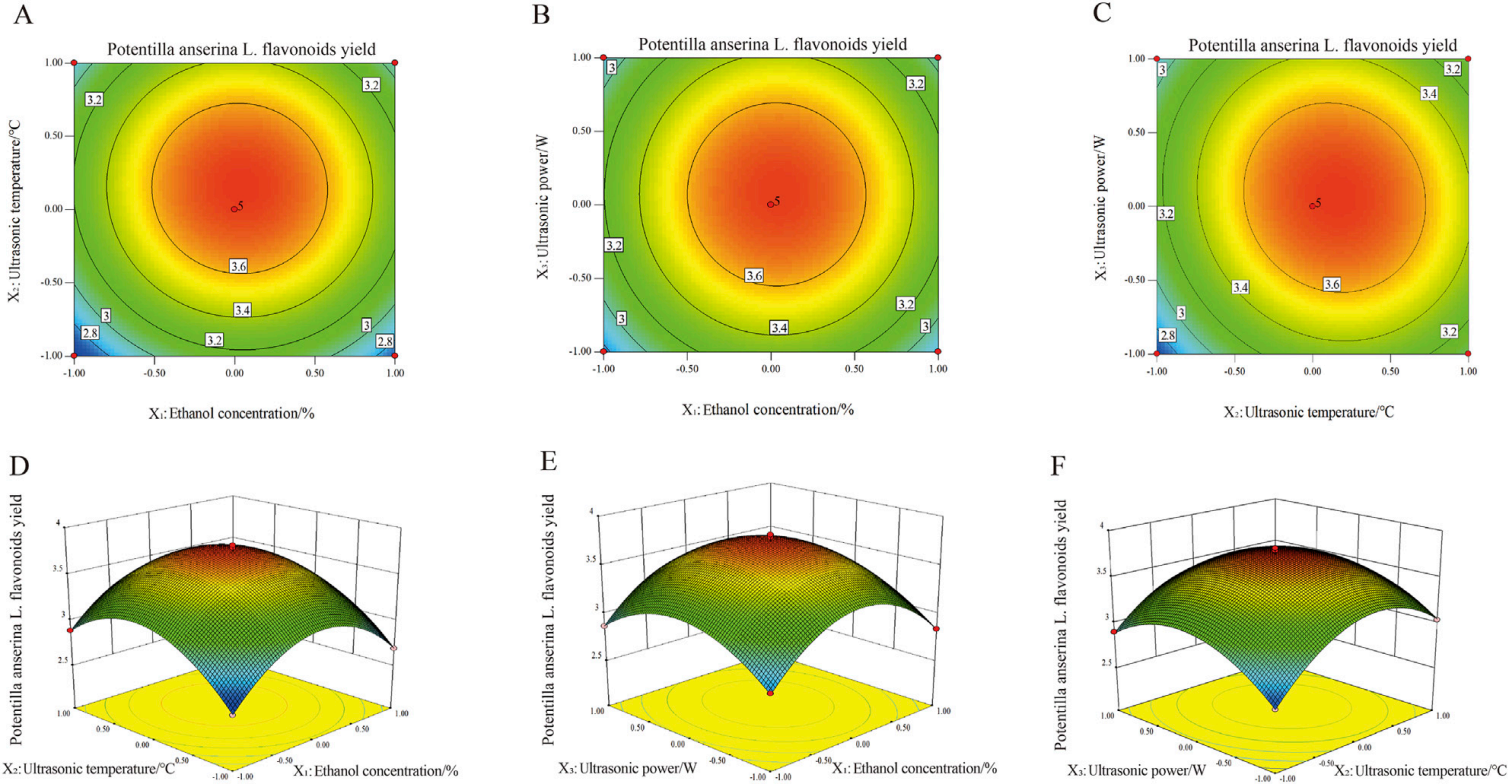
Effect of mutual interaction of independent variables on *Potentilla anserine L.* flavonoids yield (**(A–C)** represent contour maps. **(D–F)** represent 3D response surface plots).

The three-dimensional response surfaces and corresponding contour plots are presented in Figure 2. Each subplot illustrates the effects interaction of two variables on the extraction yield of *Potentilla anserina L.* flavonoids by displaying both the response surface and the contour plot. The influence of various factors on the response is hierarchical, with the intensity of the contour lines in the plots and the steepness of the response surfaces clearly reflecting the degree of impact. Closely spaced isoclines indicate a steeper response surface and a more significant effect, whereas wider intervals between isoclines suggests a relatively minor influence (Yuan et al., 2020). Among all the tested factors, ultrasonic temperature exhibited the most pronounced effect on the extraction yield of flavonoids, as evidenced by the steepest gradient observed in its 3D curve, followed by ultrasonic power and then ethanol concentration. This finding is consistent with the results of the Analysis of Variance (ANOVA).

### Validation of the predictive model

Based on Response Surface Methodology (RSM), the optimal extraction conditions for flavonoids from *Potentilla anserina L.* were determined as follows: ethanol concentration of 60.29%, ultrasonic power of 403.02 W, ultrasonic temperature of 51.4 °C, and ultrasonic time of 3 h. The theoretical yield was predicted to be 3.7554 mg/g, and the experimental obtained yield was 3.74 ± 0.06 mg/g. These results indicate that the model exhibits both good fit and predictive accuracy.

### Sephadex G-100 gel chromatography analysis

**FIGURE 3:**
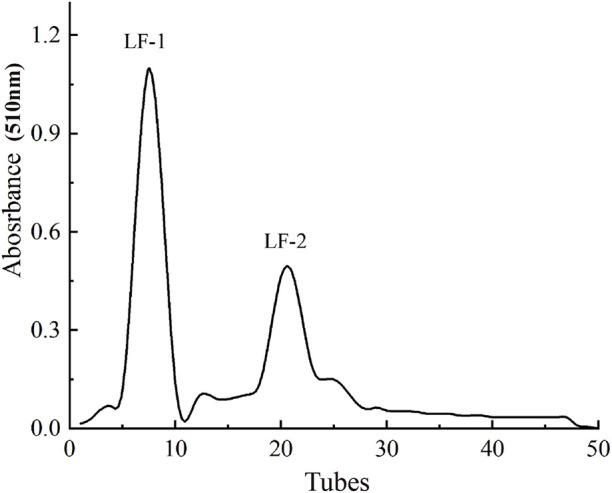
Sephadex G-100 gel chromatography.

As shown in Figure 3, after Sephadex G-100 gel chromatography, the flavonoids extracted from *Potentilla anserina L.* were preliminarily separated into two fractions based on molecular weight, designated as LF-1 and LF-2, with relative proportions of 63.34% and 25.79%, respectively. The yields of the collected fractions LF-1 and LF-2 were 41.32% and 34.56%, respectively, which represent a significant increase compared to the yields before purification.

### Antioxidant activity analysis

#### Analysis of DPPH radical scavenging activity

**FIGURE 4:**
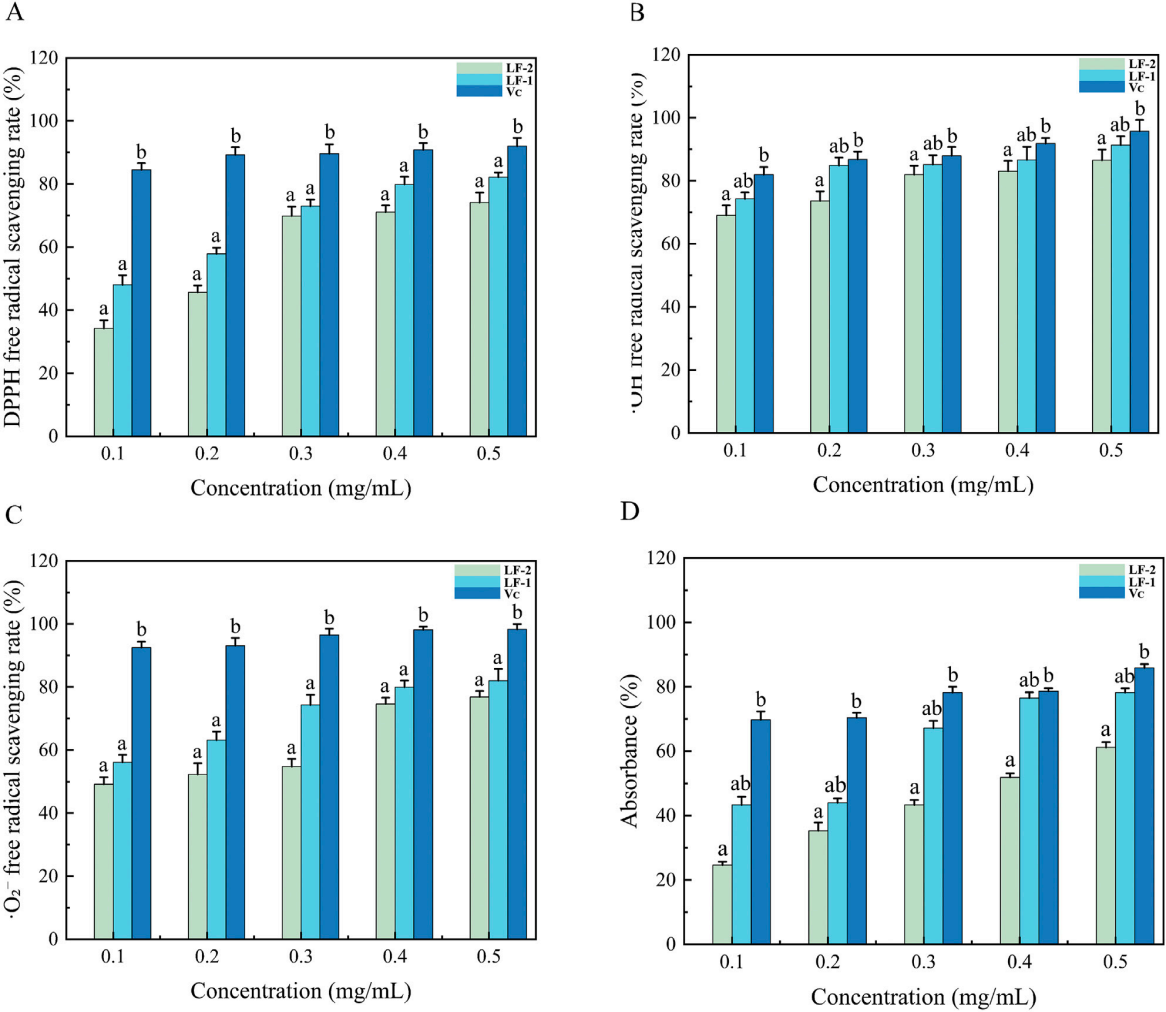
**(A)** Scavenging effect of *Potentilla anserine L.* on DPPH radicals compared with V_C_, **(B)** ·OH radicals compared with V_C_, **(C)** O_2_- radicals compared with V_C_, and **(D)**total reducing ability compared with V_C_.

Flavonoids contain multiple hydroxyl groups, most of which are capable of donating hydrogen to reduce the DPPH radical (Xu et al., 2020). The DPPH radical scavenging assay is a widely used method for the quantitative evaluation of antioxidant capacity *in vitro*. As shown in Figure 4A, the DPPH scavenging activity of LF-1 increased from 47.85% to 83.04%, and that of LF-2 increased from 33.98% to 75.55%, as the concentration rose from 0.1 to 0.5 mg/mL. This indicates that the DPPH free radical scavenging ability of flavonoids from *Potentilla anserina L.* is concentration-dependent. The reduction of the DPPH radical occurs through either hydrogen atom transfer (HAT) or single electron transfer (SET) mechanisms when antioxidants interact with it, leading to its transformation into a stable diamagnetic species (Tunnisa et al., 2022). This redox process results in a distinct hypsochromic shift in the visible spectrum of the solution, manifested as a color change from characteristic purple to pale yellow. The intensity of this color transition is quantitatively correlated with the antioxidant’s radical-neutralizing capacity of the antioxidant (Mesmar et al., 2022). Furthermore, the half-maximal inhibitory concentrations (IC_50_) were determined as 0.120 mg/mL for LF-1, 0.400 mg/mL for LF-2, and 0.11 mg/mL for the positive control ascorbic acid (V_C_). These results demonstrate that LF-1 exhibits a significant scavenging effect on DPPH free radicals.

### Analysis of hydroxyl (OH) radical scavenging activity

Hydroxyl radicals are highly reactive species that are detrimental to biological systems and capable of attacking and damaging living cells (Wang et al., 2020). Flavonoids can act as electron or hydrogen donors to neutralize hydroxyl radicals. They not only inhibit the formation of OH radicals but also scavenge existing OH radicals, demonstrating significant *in vitro* antioxidant activity (Che et al., 2023). The OH radical scavenging capacity of flavonoids from *Potentilla anserina L.* is presented in Figure 4B. Within the concentration the range of 0.1–0.5 mg/mL, the scavenging ability increased progressively. Notably, at a concentration of 0.5 mg/mL, LF-1 reached a maximum scavenging rate of 90.60% ± 0.57%, while LF-2 reached 86.15 ± 0.84%, indicating a concentration-dependent scavenging effect of *Potentilla anserina L.* flavonoids against hydroxyl radicals. The half-maximal inhibitory concentrations (IC_50_) were determined as 0.019 mg/mL for LF-1 and 0.032 mg/mL for LF-2, compared to 0.013 mg/mL for the positive control ascorbic acid (V_C_). The OH group scavenging ability of these flavonoids may be attributed to either the presence of hydroxyl groups in their molecular structures or the influence of electron density around heterocyclic carbon atoms.

### Analysis of superoxide anion (O_2_^−^) radical scavenging activity

The superoxide anion radical, a precursor of more reactive oxygen species (ROS), plays a key role in the pathogenesis of various oxidative stress-related disorders (Du et al., 2022; Lai et al., 2009). As shown in Figure 4C, flavonoids isolated from *Potentilla anserina L.* exhibited significant scavenging activity against the superoxide anion radical (·O_2_^−^). The scavenging capacity of these flavonoids increased in a concentration-dependent manner within the range of 0.1–0.5 mg/mL range. At a concentration of 0.4 mg/mL, the inhibition rates of flavonoid fractions LF-1 and LF-2 were comparable to that of the ascorbic acid (V_C_) positive control. At the maximal scavenging efficiency observed at 0.5 mg/mL, the half-maximal inhibitory concentration (IC_50_) values were determined to be 0.081 mg/mL for LF-1, 0.012 mg/mL for ascorbic acid (V_C_), and 0.139 mg/mL for LF-2. Although HF_1_ demonstrated stronger *in vitro* antioxidant activity than LF-2, its efficacy was still lower than that of V_C_. Nevertheless, LF-1 exhibited potent superoxide radical scavenging activity, indicating its potential as a functional antioxidant agent for preventing oxidative stress-induced cellular damage (Zhang et al., 2022).

### Analysis of total reducing ability

The total reducing power serves as a key indicator for assessing the redox activity of flavonoids. The reducing agent can scavenges free radicals through its inherent reductive capacity, reduce Fe^3+^ to Fe^2+^, and subsequently reacts with FeCl_3_ (Yuan et al., 2022). As shown in Figure 4D, a clear dose-dependent relationship exists between the reducing capacity and the concentration of the total flavonoid extract. Higher flavonoid concentrations correspond to stronger reducing abilities. When the concentration reaches 5 mg/mL, the reducing ability of LF-2 is most comparable to that of ascorbic acid, and the absorbance attains its maximum.

## Discussion

With the continuous improvement of modern consumers’ health awareness and ongoing advancements in food science research, concerns regarding potential health risks associated with synthetic food additives have gained increasing attention. In this context, the development of biologically safe, environmentally friendly, and highly efficacious natural-source antioxidants has become a key research focus in the field of food science and technology. This study investigates the feasibility of extracting flavonoids from *Potentilla anserina L.* for use as antioxidants, offering novel insights into the application of natural products in functional foods.

Natural products are secondary metabolites produced by organisms during evolution as adaptive responses to environmental pressures, resulting in diverse chemical structures and distinct biological functions. Flavonoids exhibit pharmacological activities—including antioxidant activity, immunomodulatory effects, anti-inflammatory properties, and cardiovascular protection—mediated by their characteristic benzene ring structures and specific hydroxyl substitution patterns. These structural features allow flavonoids to directly scavenge free radicals by donating hydrogen atoms or electrons, as well as inhibit oxidative chain reactions through metal ion chelation (Park and Han, 2022; Zhang et al., 2017). Compared with conventional extraction methods, ultrasound-assisted extraction has demonstrated advantages such as operational simplicity, shorter extraction time, higher efficiency, and the absence of a heating requirement, indicating significant potential for the extraction of bioactive components from plants materials. The cavitation effect induced by ultrasound disrupts plant cell walls, facilitates the release of flavonoids, and concurrently prevents thermal destruction of active constituents (Guo et al., 2022; Liu et al., 2022; Evary et al., 2024). In this study, the yield of flavonoids extracted from *Potentilla anserina L*. using ultrasound assistance was determined to be 3.74 ± 0.06 mg/g. This value was observed to be higher than that 2.20 ± 0.257 mg/g reported by Yan et al. for the stems and leaves of *Astragalus membranaceus* (Cui et al., 2022).

The main factors influencing the extraction yield of flavonoids include the concentration of the extraction solvent, ultrasonic temperature, ultrasonic power, and ultrasonic time. Ethanol is considered an ideal solvent for flavonoid extraction due to its dual polar and non-polar solubilization properties: its molecules can form hydrogen bonds with the polar groups of flavonoids while simultaneously dissolving non-polar structures, thereby aligning with the “like dissolves like” principle (Cui et al., 2022). Ultrasonic temperature affects extraction yield by altering solvent properties, influencing the cavitation effect, and impacting component stability. Within the range of 40 °C–60 °C, increasing temperature enhances molecular mobility; however, temperatures above 60 °C accelerate solvent volatilization and reduce the stability of cavitation bubbles, ultimately decreasing extraction efficiency (Liu et al., 2016). The impact of ultrasonic power on extraction yield follows a trend of initial increase followed by a decrease: low power facilitates cell disruption through the cavitation effect, whereas high power may lead to flavonoid degradation due to excessive heat generation. Regarding ultrasonic time, the extraction yield increases with time up to 180 min; beyond this point, the degradation rate of flavonoids surpasses the dissolution rate, resulting in no further improvement in yield (Liao et al., 2022). Response surface methodology (RSM) is utilized to model the nonlinear relationship between extraction yield and key variables such as ethanol concentration, ultrasonic time, and temperature using a multiple regression approach. This method enables efficient exploration of multi-factor combinations with fewer experimental trials, allowing precise identification of optimal conditions. The optimized extraction process determined in this study comprises an ethanol concentration of 60%, ultrasonic temperature of 50 °C, power of 400 W, and time of 180 min. The strong agreement between the model’s predicted values and the experimentally measured results confirms the effectiveness of this process optimization (Kocer et al., 2024; Md Yusof et al., 2019; Zhang et al., 2019).

High-resolution purification of flavonoids from *Potentilla anserina L.* was achieved through stepwise optimization of sample loading amount, elution gradient, and column parameters using Sephadex Gel Chromatography. Following fractionation on a Sephadex G-100 chromatography column, two distinct fractions LF-1 and LF-2 were obtained, representing 63.34% and 25.79% of the crude flavonoid content, respectively. Compared to the crude extract, the *in vitro* antioxidant activities of the purified fractions were markedly enhanced, likely due to the enrichment of active components and elimination of impurities (Zhang et al., 2024; Wang et al., 2023; Oteef, 2022). Excessive accumulation of free radicals can initiate oxidative stress cascade reactions, resulting in lipid peroxidation, organ senescence, and the development of various diseases. Supplementation with exogenous antioxidants may synergistically eliminate excessive free radicals and help restore redox homeostasis. In this study, both LF-1 and LF-2 exhibited concentration-dependent scavenging capacities against DPPH, hydroxyl, and superoxide anion radicals, along with significant total reducing power. Notably, LF-1 consistently exhibited higher *in vitro* antioxidant activity than LF-2, likely due to a higher proportion of flavonoid aglycones with ortho-diphenolic hydroxyl groups or C-ring unsaturated double bonds. Subsequently, we will perform in-depth compositional analysis (HPLC, LC-MS) to verify this for LF-1 and LF-2.

This study has demonstrated that flavonoids extracted from *Potentilla anserina L.,* particularly the LF-1 fraction, possess potent and broad-spectrum *in vitro* antioxidant activities, thereby providing a scientific basis for the high-value utilization of *Potentilla anserina L.* resources. Nevertheless, further research is required to clarify the structural characteristics of the specific active components, their *in vivo* metabolic pathways, and their stability under food industry application conditions. In future studies, flavonoid research can be conducted by using metabolomics to track their metabolic pathways in organisms, through which the patterns of active metabolites can be elucidated. This can be followed by molecular docking to analyze the binding mechanisms between metabolites and target proteins, thereby clarifying the structure-activity relationships and overcoming the limitations of focusing solely on the activity of native components.

This study is entirely based on *in vitro* experiments and has not undergone *in vivo* validation. The flavonoid components have not been subjected to in-depth characterisation via methods such as high-performance liquid chromatography (HPLC) or liquid chromatography-mass spectrometry (LC-MS). Subsequent experiments will undertake detailed chemical characterisation of the two components, LF-1 and LF-2. Findings from *in vitro* antioxidant studies cannot be directly extrapolated to physiological efficacy; animal studies will be conducted subsequently to provide *in vivo* validation.

## Conclusion

*Potentilla anserina L.* flavonoids were extracted by ultrasonic-assisted extraction. A *Potentilla anserina L.* flavonoids yield of 3.74 ± 0.06 mg/g was obtained at the optimized conditions: ethanol concentration 60%, ultrasonic power 400 W, ultrasonic temperature 50 °C, ultrasonic time 3 h. The *Potentilla anserine L.* flavonoids were preliminarily separated Sephadex G-100 chromatography, and two flavonoids’ components LF-1 and LF-2 were obtained. Between these two fractions, LF-1 was found to exhibit stronger *in vitro* antioxidant activity and significant total reducing capacity. The results suggested that *Potentilla anserine L*. flavonoids have potential application as natural antioxidant and food ingredients in functional food.

## Data Availability

The original contributions presented in the study are included in the article/supplementary material, further inquiries can be directed to the corresponding author.

## References

[B1] Binkowska I. (2020). Hesperidin: synthesis and characterization of bioflavonoid complex. SN Appl. Sci. 2 (3), 445. 10.1007/s42452-020-2256-8

[B2] Chen L. Sun J. Pan Z. Lu Y. Wang Z. Yang L. (2023). Analysis of chemical constituents of Chrysanthemum morifolium extract and its effect on postprandial lipid metabolism in healthy adults. Molecules 28 (2), 579. 10.3390/molecules28020579 36677639 PMC9866508

[B3] Chen Y. Du X.-J. Zhang Y. Liu X.-H. Wang X.-D. (2017). Ultrasound extraction optimization, structural features, and antioxidant activity of polysaccharides from Tricholoma matsutake. J. Zhejiang University-SCIENCE B 18 (8), 674–684. 10.1631/jzus.B1600239 28786242 PMC5565515

[B4] Chen H. Shi X. Zhang L. Yao L. Cen L. Li L. (2022). Ultrasonic extraction process of polysaccharides from Dendrobium nobile Lindl.: optimization, physicochemical properties and anti-inflammatory activity. Foods 11 (19), 2957. 10.3390/foods11192957 36230031 PMC9564065

[B5] Čižmárová B. Hubková B. Tomečková V. Birková A. (2023). Flavonoids as promising natural compounds in the prevention and treatment of selected skin diseases. Int. J. Mol. Sci. 24 (7), 6324. 10.3390/ijms24076324 37047297 PMC10094312

[B6] Cui L. Ma Z. Wang D. Niu Y. (2022). Ultrasound-assisted extraction, optimization, isolation, and antioxidant activity analysis of flavonoids from Astragalus membranaceus stems and leaves. Ultrason. Sonochemistry 90, 106190. 10.1016/j.ultsonch.2022.106190 36215890 PMC9554832

[B7] Du T. Xu J. Zhu S. Yao X. Guo J. Lv W. (2022). Effects of spray drying, freeze drying, and vacuum drying on physicochemical and nutritional properties of protein peptide powder from salted duck egg white. Front. Nutr. 9, 1026903. 10.3389/fnut.2022.1026903 36337632 PMC9626763

[B8] Evary Y. M. Alam G. Raihan M. Khotimah K. (2024). Extraction of flavonoids from parasitic plant Macrosolen cochinchinensis using ultrasound-assisted extraction: an optimization approach. J. Exp. Biol. Agric. Sci. 12 (4), 616–624. 10.18006/2024.12(4).616.624

[B9] Franco E. P. D. d. Contesini F. J. Lima da Silva B. Alves de Piloto Fernandes A. M. Wielewski Leme C. Gonçalves Cirino J. P. (2019). Enzyme-assisted modification of flavonoids from matricaria chamomilla: antioxidant activity and inhibitory effect on digestive enzymes. J. Enzyme Inhibition Med. Chem. 35 (1), 42–49. 10.1080/14756366.2019.1681989 31656110 PMC6830229

[B10] Goswami S. Das R. Ghosh P. Chakraborty T. Barman A. Ray S. (2020). Comparative antioxidant and antimicrobial potentials of leaf successive extract fractions of poison bulb, *Crinum asiaticum* L. Industrial Crops Prod. 154, 112667. 10.1016/j.indcrop.2020.112667

[B11] Guo L. Kong N. Zhang X. Ma H. (2022). Multimode ultrasonic extraction of polysaccharides from maca (lepidium meyenii): optimization, purification, and *in vitro* immunoregulatory activity. Ultrason. Sonochemistry 88, 106062. 10.1016/j.ultsonch.2022.106062 35751935 PMC9240871

[B12] Guo P. Chen H. Ma J. Zhang Y. Chen H. Wei T. (2023). Enzyme-assisted extraction, characterization, and *in vitro* antioxidant activity of polysaccharides from Potentilla anserina L. Front. Nutr. 10, 1216572. 10.3389/fnut.2023.1216572 37528998 PMC10388540

[B13] Huang Y. Guo J. Zhang J. (2020). Physicochemical and antioxidant properties of Potentilla anserina L. polysaccharides affected by ultrasonication. Appl. Sci. 10 (13), 4510. 10.3390/app10134510

[B14] Kim T. Y. Leem E. Lee J. M. Kim S. R. (2020). Control of reactive oxygen species for the prevention of parkinson’s disease: the possible application of flavonoids. Antioxidants 9 (7), 583. 10.3390/antiox9070583 32635299 PMC7402123

[B15] Kocer S. Utku Copur O. Ece Tamer C. Suna S. Kayahan S. Uysal E. (2024). Optimization and characterization of chestnut shell pigment extract obtained microwave assisted extraction by response surface methodology. Food Chem. 443, 138424. 10.1016/j.foodchem.2024.138424 38301551

[B16] Lai J.-T. Wang W.-C. Liao C.-M. Huang T.-C. Liao C.-C. (2009). Cultivation of Aspergillus phoenicis with high superoxide dismutase-like activity. J. Taiwan Inst. Chem. Eng. 40 (5), 485–490. 10.1016/j.jtice.2009.02.005

[B17] Li W. Wang Y. Wei H. Zhang Y. Guo Z. Qiu Y. (2020). Structural characterization of Lanzhou lily (Lilium davidii var. unicolor) polysaccharides and determination of their associated antioxidant activity. J. Sci. Food Agric. 100 (15), 5603–5616. 10.1002/jsfa.10613 32608519

[B18] Liang Y. Farooq M. U. Hu Y. Tang Z. Zhang Y. Zeng R. (2018). Study on stability and antioxidant activity of red anthocyanidin glucoside rich hybrid rice, its nutritional and physicochemical characteristics. Food Sci. Technol. Res. 24 (4), 687–696. 10.3136/fstr.24.687

[B19] Liao J. Guo Z. Yu G. (2021). Process intensification and kinetic studies of ultrasound-assisted extraction of flavonoids from peanut shells. Ultrason. Sonochemistry 76, 105661. 10.1016/j.ultsonch.2021.105661 34252684 PMC8343109

[B20] Liao J. Xue H. Li J. (2022). Extraction of phenolics and anthocyanins from purple eggplant peels by multi-frequency ultrasound: effects of different extraction factors and optimization using uniform design. Ultrason. Sonochemistry 90, 106174. 10.1016/j.ultsonch.2022.106174 36170772 PMC9513698

[B21] Liu J.-L. Li L.-Y. He G.-H. (2016). Optimization of microwave-assisted extraction conditions for five major bioactive compounds from Flos sophorae immaturus (cultivars of Sophora japonica L.) using response surface methodology. Molecules 21 (3), 296. 10.3390/molecules21030296 26950107 PMC6274464

[B22] Liu L. Luo P. Xiao J. (2019). Effect of carboxymethylation and phosphorylation on the properties of polysaccharides from Sepia esculenta ink: antioxidation and anticoagulation *in vitro* . Mar. Drugs 17 (11), 626. 10.3390/md17110626 31683929 PMC6891342

[B23] Liu Y. Zhang D. Li X. Xiao J. Guo L. (2022). Enhancement of ultrasound-assisted extraction of sulforaphane from broccoli seeds via the application of microwave pretreatment. Ultrason. Sonochemistry 87, 106061. 10.1016/j.ultsonch.2022.106061 35716467 PMC9213254

[B24] Luan G. Yang M. Nan X. Lv H. Liu Q. Wang Y. (2022). Optimization and comparative study of different extraction methods of sixteen fatty acids of Potentilla anserina L. from twelve different producing areas of the Qinghai-Tibetan Plateau. Molecules 27 (17), 5443. 10.3390/molecules27175443 36080209 PMC9457940

[B25] Md Yusof A. H. Abd Gani S. S. Zaidan U. H. Halmi M. I. E. Zainudin B. H. (2019). Optimization of an ultrasound-assisted extraction condition for flavonoid compounds from cocoa shells (Theobroma cacao) using response surface methodology. Molecules 24 (4), 711. 10.3390/molecules24040711 30781448 PMC6412431

[B26] Mesmar J. Abdallah R. Hamade K. Baydoun S. Al-Thani N. Shaito A. (2022). Ethanolic extract of Origanum syriacum L. leaves exhibits potent anti-breast cancer potential and robust antioxidant properties. Front. Pharmacol. 13, 994025. 10.3389/fphar.2022.994025 36299882 PMC9589507

[B27] Michalaki A. Karantonis H. C. Kritikou A. S. Thomaidis N. S. Dasenaki M. E. (2023). Ultrasound-assisted extraction of total phenolic compounds and antioxidant activity evaluation from oregano (Origanum vulgare ssp. hirtum) using response surface methodology and identification of specific phenolic compounds with HPLC-PDA and Q-TOF-MS/MS. Molecules 28 (5), 2033. 10.3390/molecules28052033 36903279 PMC10004109

[B28] Nie Z. Wan C. Chen C. Chen J. (2019). Comprehensive evaluation of the postharvest antioxidant capacity of Majiayou Pomelo harvested at different maturities based on PCA. Antioxidants 8 (5), 136. 10.3390/antiox8050136 31108913 PMC6563022

[B29] Oteef M. D. Y. (2022). Comparison of different extraction techniques and conditions for optimizing an HPLC-DAD method for the routine determination of the content of chlorogenic acids in green coffee beans. Separations 9 (12), 396. 10.3390/separations9120396

[B30] Park J.-E. Han J.-S. (2022). HM-Chromanone, a major homoisoflavonoid in Portulaca oleracea L., improves palmitate-induced insulin resistance by regulating phosphorylation of IRS-1 residues in L6 skeletal muscle cells. Nutrients 14 (18), 3815. 10.3390/nu14183815 36145191 PMC9504146

[B31] Tang C. Li X. Chen J. Liang J. Wang T. Li Y. (2022). Characterization and phylogenetic relationship of the complete chloroplast genome of a Chinese traditional medicinal plant Potentilla anserina L. Mitochondrial DNA Part B 7 (9), 1653–1655. 10.1080/23802359.2022.2119817 36147363 PMC9487938

[B32] Tang Z. Wang Y. Huang G. Huang H. (2023). Ultrasound-assisted extraction, analysis and antioxidant activity of polysaccharide from the rinds of Garcinia mangostana L. Ultrason. Sonochemistry 97, 106474. 10.1016/j.ultsonch.2023.106474 37321072 PMC10311166

[B33] Tunnisa F. Nur Faridah D. Afriyanti A. Rosalina D. Ana Syabana M. Darmawan N. (2022). Antioxidant and antidiabetic compounds identification in several Indonesian underutilized Zingiberaceae spices using SPME-GC/MS-based volatilomics and *in silico* methods. Food Chem. X 14, 100285. 10.1016/j.fochx.2022.100285 35342880 PMC8943257

[B34] Wang M. Lei M. Samina N. Chen L. Liu C. Yin T. (2020). Impact of Lactobacillus plantarum 423 fermentation on the antioxidant activity and flavor properties of rice bran and wheat bran. Food Chem. 330, 127156. 10.1016/j.foodchem.2020.127156 32531631

[B35] Wang Y.-F. Lin P. Huang Y.-L. He R.-J. Yang B.-Y. Liu Z.-B. (2023). Isolation of two new phenolic glycosides from Castanopsis chinensis hance by combined multistep CC and HSCCC separation and evaluation of their antioxidant activity. Molecules 28 (8), 3331. 10.3390/molecules28083331 37110565 PMC10143513

[B36] Wu M. Liu P. Wang S. Zhong C. Zhao X. (2021). Ultrasonic microwave-assisted micelle combined with fungal pretreatment of Eucommia ulmoides leaves significantly improved the extraction efficiency of total flavonoids and gutta-percha. Foods 10 (10), 2399. 10.3390/foods10102399 34681448 PMC8535202

[B37] Xu M. Shen C. Zheng H. Xu Y. Xue C. Zhu B. (2020). Metabolomic analysis of acerola cherry (Malpighia emarginata) fruit during ripening development via UPLC-Q-TOF and contribution to the antioxidant activity. Food Res. Int. 130, 108915. 10.1016/j.foodres.2019.108915 32156365

[B38] Xue Y. Wang F. Zhou C. (2022). Optimization of ultrasonic extraction of triterpenes from loquat peel and pulp and determination of antioxidant activity and triterpenoid components. Foods 11 (17), 2563. 10.3390/foods11172563 36076748 PMC9455252

[B39] Xue H. Li J. Wang G. Zuo W. Zeng Y. Liu L. (2022). Ultrasound-assisted extraction of flavonoids from Potentilla fruticosa L. using natural deep eutectic solvents. Molecules 27 (18), 5794. 10.3390/molecules27185794 36144529 PMC9504222

[B40] Yang D. Wang L. Zhai J. Han N. Liu Z. Li S. (2021). Characterization of antioxidant, α-glucosidase and tyrosinase inhibitors from the rhizomes of Potentilla anserina L. and their structure–activity relationship. Food Chem. 336, 127714. 10.1016/j.foodchem.2020.127714 32828014

[B41] Yu G. Yu X. Yang G. Tang Y. Diao Y. (2018). A novel diagnostic method to detect duck tembusu virus: a colloidal gold-based immunochromatographic assay. Front. Microbiol. 9, 1001. 10.3389/fmicb.2018.01001 29867893 PMC5963251

[B42] Yuan S. Xu C. Y. Xia J. Feng Y. N. Zhang X. F. Yan Y. Y. (2020). Extraction of polysaccharides from Codonopsis pilosula by fermentation with response surface methodology. Food Sci. and Nutr. 8 (12), 6660–6669. 10.1002/fsn3.1958 33312549 PMC7723197

[B43] Yuan T. Huang J. Gan L. Chen L. Zhong J. Liu Z. (2022). Ultrasonic enhancement of aqueous two-phase extraction and acid hydrolysis of flavonoids from Malvaviscus arboreus cav. Flower for evaluation of antioxidant activity. Antioxidants 11 (10), 2039. 10.3390/antiox11102039 36290762 PMC9598477

[B44] Zhang X. Li H. Zhang H. Liu Y. Huo L. Jia Z. (2017). Inhibition of transmembrane member 16A calcium‐activated chloride channels by natural flavonoids contributes to flavonoid anticancer effects. Br. J. Pharmacol. 174 (14), 2334–2345. 10.1111/bph.13841 28452066 PMC5481650

[B45] Zhang Q.-W. Lin L.-G. Ye W.-C. (2018). Techniques for extraction and isolation of natural products: a comprehensive review. Chin. Med. 13 (1), 20. 10.1186/s13020-018-0177-x 29692864 PMC5905184

[B46] Zhang L. Jiang Y. Pang X. Hua P. Gao X. Li Q. (2019). Simultaneous optimization of ultrasound-assisted extraction for flavonoids and antioxidant activity of Angelica keiskei using response surface methodology (RSM). Molecules 24 (19), 3461. 10.3390/molecules24193461 31554203 PMC6804174

[B47] Zhang Y. Bian S. Hu J. Liu G. Peng S. Chen H. (2022). Natural deep eutectic solvent-based microwave-assisted extraction of total flavonoid compounds from spent sweet potato (Ipomoea batatas L.) leaves: optimization and antioxidant and bacteriostatic activity. Molecules 27 (18), 5985. 10.3390/molecules27185985 36144716 PMC9501105

[B48] Zhang D. Xiao D. Yin T. Zhao S. Zhur O. Xiao X. (2024). The extraction of effective components and an antioxidant activity study of Tulipa edulis. Food Sci. Hum. Wellness 13 (1), 276–286. 10.26599/fshw.2022.9250023

[B49] Zhou S. Huang G. Chen G. (2021). Extraction, structural analysis, derivatization and antioxidant activity of polysaccharide from Chinese yam. Food Chem. 361, 130089. 10.1016/j.foodchem.2021.130089 34029907

